# Incorporating equity, diversity and inclusion (EDI) into the education and assessment of professionalism for healthcare professionals and trainees: a scoping review

**DOI:** 10.1186/s12909-024-05981-3

**Published:** 2024-09-11

**Authors:** Darsh Shah, Nima Behravan, Nujud Al-Jabouri, Matthew Sibbald

**Affiliations:** 1 Michael G. DeGroote School of Medicine, McMaster University, Hamilton, ON Canada; 2 Faculty of Health Sciences, McMaster University, Hamilton, ON Canada

**Keywords:** Scoping review, Professionalism, EDI, Cultural humility, Advocacy, Health professions education

## Abstract

**Background:**

Current definitions of professionalism for healthcare trainees often lack equity, diversity and inclusion (EDI) in the expectations and assessment of professionalism. While professionalism teaching is incorporated in healthcare training, equity-deserving groups still experience discrimination. This scoping review investigates the literature to understand how EDI and associated domains of cultural humility, and advocacy can be incorporated in healthcare trainees’ education and assessment of professionalism.

**Methods:**

The Arksey and O’Malley framework was applied to this scoping review. MEDLINE, Embase & PsychINFO were searched up to March 2023, with terms surrounding health professionals, professionalism, EDI, cultural humility, and advocacy. Titles and abstracts (*n* = 3870) and full-texts (*n* = 140) were independently screened by two reviewers. Articles were included if they focused on EDI, cultural humility, or advocacy among healthcare students/trainees, and had outcomes related to professionalism. Articles lacking discussion of professionalism as an outcome were excluded. Themes were generated by mutual discussion. Risk of bias was assessed using the Cote et al. and Medical Education Research Study Quality Instrument (MERSQI) tools.

**Results:**

48 articles underwent thematic analysis. Studies investigated the disciplines of medicine, nursing, social work, physiotherapy, and dentistry. Most studies were qualitative in methodology (*n* = 23). Three themes emerged: (1) EDI-related interventions are associated with improved professionalism of healthcare trainees/workers (*n* = 21). Interventions employed were either an EDI-associated educational course (*n* = 8) or an exchange program to promote EDI competencies among trainees (*n* = 13). (2) Trainee definitions and perceptions of professionalism include themes related to EDI and cultural humility (*n* = 12). (3) Current standards of professionalism are perceived as non-inclusive towards historically-marginalized populations (*n* = 15). Literature investigating advocacy as it relates to professionalism is limited.

**Conclusion:**

This review identified that core EDI principles and its associated domains of cultural humility and advocacy are often viewed as integral to professionalism. These findings create a strong impetus to incorporate EDI principles within professionalism frameworks in healthcare education. Future research should employ standardized tools for professionalism assessment to provide more conclusive evidence. Incorporating patient perspectives of professionalism can inform actionable recommendations for fostering inclusive healthcare environments.

**Supplementary Information:**

The online version contains supplementary material available at 10.1186/s12909-024-05981-3.

## Introduction

In the healthcare pedagogical literature, professionalism has often been broadly defined as a set of characteristics, competencies and attitudes that are expected of a healthcare professional or trainee [1]. A paper published in the American Medical Association Journal of Ethics suggests that upholding equity is amongst the most important roles of a physician by stating: “Organizational, system, and policy reform demand that professionalism be redefined in terms of its capacity to motivate equity in health professions education and clinical practice” [2]. However, despite attempts of healthcare institutions to define specific domains and competencies in their professionalism frameworks, concepts of EDI, cultural humility and advocacy are often omitted in these frameworks [2].

An example of a healthcare professionalism framework is one developed by the Michael G. DeGroote School of Medicine (MGDSM) at McMaster University in Hamilton, Ontario, Canada. This framework is called the Professionalism in Practice (PIP) framework, which teaches and holds its learners accountable to the following core domains of professionalism: professional responsibility and integrity, pursuit of excellence/insight, personal interactions, as well as EDI and Indigenous reconciliation [3]. As noted here, the MGDSM makes a deliberate effort to outline EDI and Indigenous reconciliation as a core domain to uphold as a standard of its learners’ and providers’ professionalism. Another example of a healthcare professionalism framework is that developed by the National Taiwan University College of Medicine (NTUCM). The NTUCM developed this medical professionalism framework through contributions from stakeholders, including chairs of hospital departments, residents, and attending physicians. The resultant consensus framework consisted of 8 domains: integrity, humanism, altruism, communication, clinical competence, ethics, excellence, and accountability [4]. Despite some overlap between the healthcare professionalism frameworks of NTUCM and MGDSM, various competencies are not shared across the two frameworks. For example, the NTUCM framework does not explicitly have a domain pertinent to EDI, cultural humility or advocacy [4]. Therefore, due to the inherent subjectivity and lack of unison in the definitions and domains of professionalism frameworks at different institutions, crucial concepts such as EDI, cultural humility and advocacy may be left out of professionalism curricula and frameworks.

Numerous studies find that EDI, cultural humility and advocacy are beneficial for both patients and healthcare teams. A review paper found that multiple studies suggest patient outcomes are improved by more diverse teams, and that healthcare environments that are identified as diverse are found to be less prone to disputes in times of change [5]. Additionally, despite the efforts of healthcare institutions in teaching, assessing and upholding professionalism expectations from their trainees, discriminatory practices and consequent healthcare disparities still persist. For instance, in Canada, Black and Indigenous communities continue to experience discrimination when seeking healthcare services [6–10]. In an ethnographic study conducted in an emergency department (ED) in a large teaching hospital in a Western Canadian city, it was found that many Indigenous patients felt they were being judged on the basis of their identity, and that presumptions were made that their visits to the ED were due to illegitimate pain issues or inappropriate reasons [6]. An important example of discriminatory healthcare practices against Indigenous communities is that of Joyce Echaquan, an Indigenous patient at a Quebec hospital who, moments before her passing, recorded a video displaying her screaming in pain while her healthcare providers made distasteful remarks towards her based on racially-charged stereotypes [7]. Additionally, a qualitative study on self-identified Black individuals who lived in Montréal during the COVID-19 pandemic found several themes regarding internalized anti-Black racism amongst healthcare providers, including the presence of insensitivity towards racial discrimination by some providers [8].

The discriminatory practices and healthcare disparities amongst patients of equity-deserving backgrounds noted above [6–10], combined with the lack of emphasis on EDI in healthcare professionalism definitions [2], creates a strong impetus for redefining expectations of professionalism from healthcare professionals and trainees to encompass concepts of cultural humility and advocacy.

This scoping review systematically searches the literature to evaluate how EDI and associated domains of cultural humility and advocacy are related to and can be incorporated into the standards, teaching and evaluation of professionalism for healthcare workers and trainees.

## Methods

### Research question

This scoping review aggregates the existing evidence on the following research question: *What literature is available on how principles of equity*,* diversity and inclusion (EDI) are incorporated in the education and assessment of professionalism for health professions trainees?*

### Approach to terminology

This paper utilizes the terminology recommended by the American Medical Association (AMA) and the Association of American Medical Colleges (AAMC) to guide language, narrative, and concepts as our standard for inclusive language that promotes health equity. For instance, per the AMA and AAMC recommendations, the terms “cultural competency”, “minority/minority groups” and “equality” as commonly found in the literature, are substituted with terms such as “cultural humility/safety”, “(people from) racial and ethnic groups’’ and “equity”, respectively [11].

## Materials and methods

We adopted scoping review methodology to understand the breadth and depth of literature pertaining to the principles of EDI in professionalism education and assessment [12]. We were guided by Arksey and O’Malley’s methodological framework for scoping reviews, which includes five main stages (1) identifying the research question; (2) identifying relevant studies; (3) study selection; (4) charting the data; and (5) collating, summarizing and reporting the results [13]. We reported our process according to the Preferred Reporting Items for Systematic Reviews and Meta-Analyses extension for Scoping Reviews (PRISMA-ScR) Checklist through multiple rounds of feedback and modification by our research team [14].

### Eligibility criteria

Studies were included if (1) the population was practicing health professionals and trainees working in the fields of medicine, nursing, social work, rehabilitation, multidisciplinary healthcare teams, medical education, dentistry, midwifery, and pharmacy (2) the outcome was related to professionalism within the objectives, methodology, or results sections, and (3) professionalism was linked to EDI, cultural humility, or advocacy. Studies were excluded if they (1) were not in English, (2) did not focus on professionalism outcomes, (3) did not focus on our population of interest, and (4) were grey literature publications (e.g. conference proceedings, abstracts, non-peer reviewed reports). Considering that both empirical and non-empirical studies have the potential to yield meaningful findings on concepts of EDI and professionalism, no restrictions were placed on study design.

### Search strategy

A preliminary search was conducted to identify keywords and subject headings related to our research question. The final search strategy was developed in consultation among the research team members and a librarian at McMaster Health Sciences Library. Three electronic databases were searched from inception to March 7, 2023: Embase, Medline and PsycINFO. Search terms were designed to capture concepts of “advocacy” AND “cultural humility” AND “healthcare professionals” AND “healthcare trainees” AND “professionalism” AND “professional development.” Search terms were adapted for each database as subject headings or keywords where appropriate (See Appendix S1, Additional File 1). To identify any non-indexed literature, we hand searched the reference list of included studies and Google Scholar. The search strategy used the same search term combinations as described above and was limited to literature published in peer-reviewed journals only. Grey literature, such as individual institution’s professionalism frameworks, were not included. Covidence software was used to manage citations, including removing duplicates and screening [15].

A revised search was conducted between March 7th 2023 to April 1st 2024 with an identical search strategy to identify advances in the literature. A set of 204 unique records were identified for which abstract and full-text screening were completed. We did not identify any new studies meeting inclusion/exclusion criteria that added substantial evidence to the results of our study.

### Screening process

**Fig. 1:**
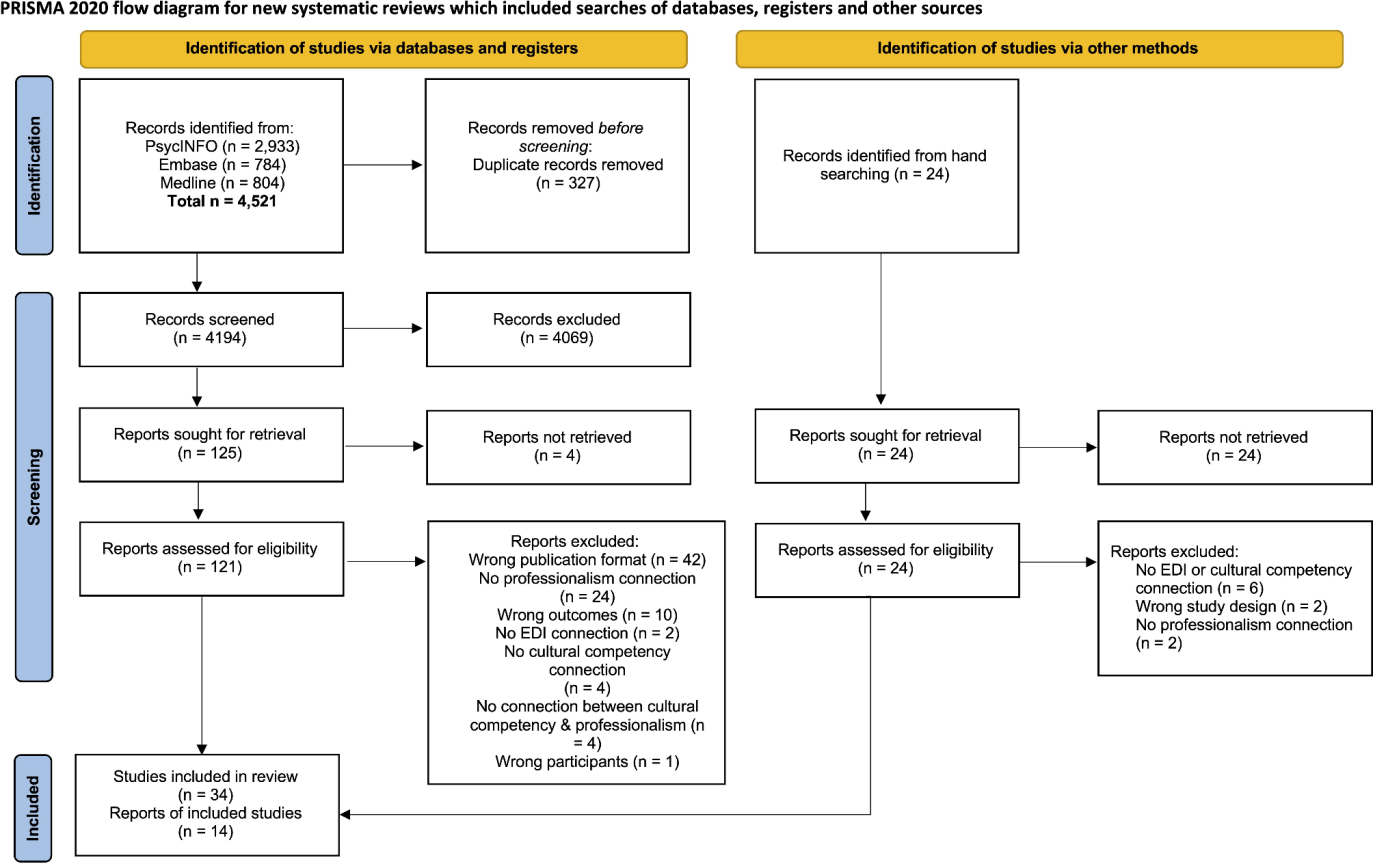
PRISMA-ScR Flow Diagram

A pilot screening test of the eligibility criteria was conducted by two independent reviewers (NA, DS, or NB) on a small sample of studies. Reviewers met to discuss their agreement level and the eligibility criteria was modified for clarity. Following this pilot test, two independent reviewers (NA, DS, or NB) screened titles and abstracts to evaluate their eligibility against the inclusion and exclusion criteria. Studies that passed the initial screening process underwent full-text screening by two independent reviewers (NA, DS, or NB). Disagreements at any screening stage were resolved by a third reviewer or consensus-based discussion. A PRISMA-ScR flowchart was used to show the process of study selection (Fig. 1).

### Data extraction

A data extraction sheet was developed by the authors and used to organize data from included studies. Data extraction was divided among three members (NA, DS or NB) and was conducted using the Excel software. All extracted data was reviewed by a different member (NA, DS or NB). The final extraction table included (1) study characteristics (i.e., authors, publication year, country), (2) sample characteristics (i.e., number of participants, population description), (3) methodological characteristics (i.e., study design, program description/recruitment), and (4) study outcomes related to professionalism (See Appendix S2, Additional File 1).

### Quality appraisal

One reviewer (NA, DS or NB) assessed the quality of studies using tools for qualitative and quantitative data. This consisted of the Medical Education Research Study Quality Instrument (MERSQI) for quantitative studies, and a qualitative study grid published by Côté et al. [16, 17].

### Data analysis

This review qualitatively analyzed the data following the thematic analysis approach [18]. Three reviewers (NA, DS & NB) independently categorized data according to their meaning and content. Subsequently, key themes were independently identified and formulated. These themes were then compared and any disagreements were resolved with discussion-based consensus, arriving at three main themes.

## Results

### Literature scope and characteristics

Results of the initial search are presented in Fig. 1, including rationales for excluded articles. The data searches yielded 4194 unique records, with 125 selected for full-text review. After screening against eligibility criteria, 34 articles were included for data extraction. Included articles spanned publication between 2005 and 2023. An additional 24 articles were identified from hand-searching of included studies, of which 14 met eligibility criteria, producing a total of 48 articles included in our analysis.

### Literature scope and characteristics

Most studies involved medical trainees or physicians (*n* = 18). Other professions examined in the literature included nursing, social work, dentistry, midwifery, and physical therapists. Most studies were based in the United States (*n* = 19) among other countries such as Australia (*n* = 4), the United Kingdom (*n* = 1), Canada (*n* = 4), and Turkey (*n* = 3) with eleven studies being multinational (*n* = 11). The majority of studies were qualitative in nature (*n* = 23) followed by mixed-methods (*n* = 10), commentaries (*n* = 8), and quantitative (*n* = 7). Characteristics of the studies divided by themes can be found in the supplementary material (Appendix S2, Additional File 1) Among qualitative studies, methods of evaluating professionalism were predominantly based on participant interviews (*n* = 16) followed by surveys (*n* = 13), written narratives (*n* = 9), and literature review (*n* = 3). Quantitative measures included internally designed Likert scales to measure participants’ perspectives (*n* = 14) with few studies employing validated tools (*n* = 3). Twenty-one studies employed an EDI training intervention, split between educational courses (*n* = 8) and clinical placements (*n* = 11). A summary of quality appraisal is presented in the supplementary material (See Appendix S3, Additional File 1).

#### Theme 1: EDI-related interventions are associated with improved professionalism of healthcare trainees/professionals

Most studies investigated outcomes after an EDI-oriented intervention on professionalism outcomes among participants (*n* = 21). Studies within this theme are divided based on their intervention of an educational course (*n* = 8) or an exchange clinical placement (*n* = 13). Topics of the educational courses included health advocacy, cultural humility, and spirituality/religion [19–26]. All eight studies reported an improvement in professionalism or professional identity formation. Four of the eight studies explicitly link professionalism and EDI concepts [19, 20, 22, 26]. In one study, first-year medical students completed a 10-month community health elective course aimed to expand cultural humility and advocacy in the context of adolescent care [22]. Reflective essays completed at the end of the course revealed that 90% of students noted an increase in knowledge and skills of professionalism [22]. These findings are seen across healthcare disciplines including nursing, pharmacy, social work, and other allied health. A 10-week interprofessional course aimed at developing cultural humility induced professional growth in addition to improved cultural proficiency among nursing, pharmacy, and social work trainees [26]. These studies demonstrate improved professionalism as an outcome for EDI-promotion through healthcare education.

Studies employing clinical placement interventions as methods of EDI-training revolved around either rural community or international placements [27–39]. Professionalism outcomes included a heterogenous combination of reflections on patient-clinican and interprofessional interactions [32, 34]. All thirteen studies report improvement in domains of professionalism secondary to exchange programs within different cultural settings [27–39]. A 4-week exchange between Japanese and UK medical students was associated with professionalism outcomes including social justice and resource stewardship [38]. Five of the thirteen studies explicitly connect domains of EDI to professionalism or professional development [28, 29, 33, 36, 37]. In these cases, cross-cultural experiences directly improve professionalism in participants [31, 33, 36]. Remaining studies describe parallel improvements in cultural humility and professionalism as outcomes of the intervention with implicit associations between the two concepts [27, 39].

#### Theme 2: operationalization of professionalism revealed themes of EDI

A second subset of studies assessed trainee conceptualization of health professionalism within their respective field of practice (*n* = 12) [40–51]. These studies investigate the attitudes, perspectives, and competencies of trainees on professionalism in relationship to domains of EDI. Trainees note that adaptibility and humility are critical elements of professionalism, which extends to adapting to cultural and social norms [51]. Inability to accomodate differences in gender norms, language, or cultural beliefs are sources of professionalism dilemmas [44]. Hamdan Alshammari and Alboliteeh applied a structural equation model to questionnaire responses of 587 nurses in Saudi Arabia. They find significant correlations between dimensions of professionalism and cultural competency [50]. Similarly, practitioner perceptions of cultural competency are closely related to perceptions of professional development rather than formal health professional education [43]. A bidirectional relationship between professionalism and themes of EDI is demonstrated in the literature. Topics of EDI including cultural humility and health advocacy are often cited as core components of professional development [47, 51]. Simultaneously, medical trainees, physicians, and researchers identify a need to integrate professionalism assessment within the development of an effective cultural humility curricula [46]. Conventional professionalism values such patient centeredness, bias recognition and clinical skills are necessary for cultural humility [46]. Therefore, not only is EDI training a contributing factor to professionalism, professional identity development is required for acquisition of EDI competencies.

#### Theme 3: current standards of professionalism are perceived as non-inclusive

**Fig. 2:**
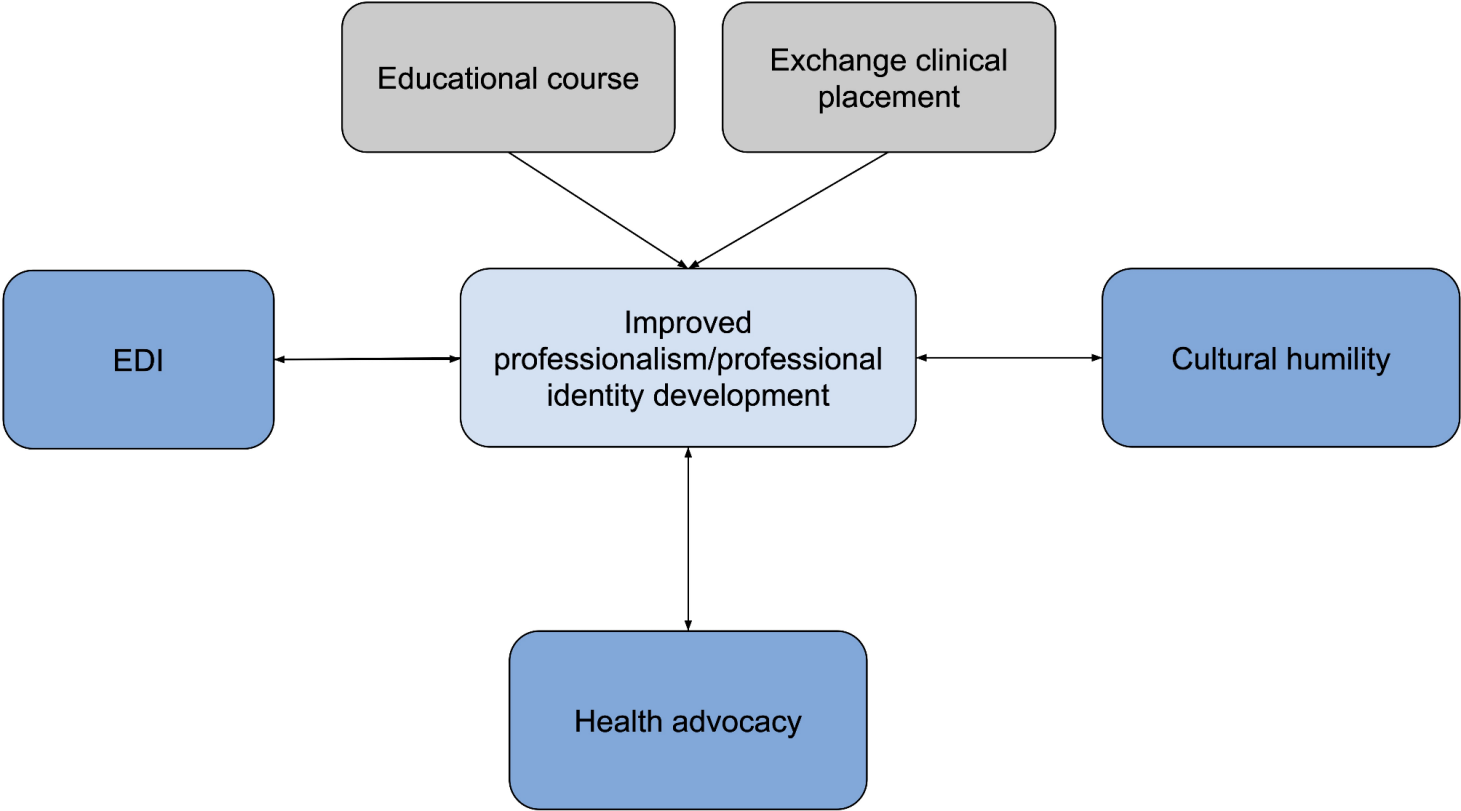
Integrated model of professionalism including EDI, cultural humility, and health advocacy

The final set of studies explored the perspectives of trainees and practitioners from equity-deserving groups (n= [52–66]. Specifically, researchers investigated perceptions of professionalism in the workplace with reference to gender identity, sexual orientation, race/ethnicity and other EDI-associated demographic factors [52, 53]. Participants from equity-deserving groups routinely experience professionalism as culturally and ethnically restrictive [53]. In these studies, professionalism is defined as a reflection of the cultural norms and expectations of the dominant social group [59]. Given the historical context in which professionalism was established, these norms often stem from the “White male identity” [53]. Consequently, trainees and practitioners that deviate from existing definitions of professionalism in terms of racial or ethnic identity, culture, skin colour, gender identity, sexual orientation, or colour are at a disadvantage. Survey of practitioners from equity-deserving backgrounds notes increased criticism over professional actions and increased pressure to conform. Furthermore, cultural and ethnic incongruity with the professionalism standard inhibits professional development to positions of leadership [54, 55]. This literature is also surrounded by a handful of recent commentaries by physicians on the current inequities faced by equity-deserving populations [59–65]. Rosenberg and colleagues discuss three cases in which professionalism standards propagated disparities within medicine. One case is of a Latin medical student who was deemed unprofessional in a clinical examination as she was wearing Latin earrings [61]. Dr. AbdelHameid shares her experiences as a Black physician burden by an expectation to comply with racially charged interactions with patients and colleagues in order to align with professionalism expectations [62]. International medical graduates highlight the unique challenges of adapting to professionalism norms due to the inherent interconnectedness of professional identity and cultural schemata [65]. A common motif remains a sense of rigidity in definitions of professionalism with limited ethnic expression afforded to practitioners [59, 62]. Accordingly, there is increasing support for the existing definitions of professionalism to include measures of equity, diversity, and cultural humility [52, 55, 56]. Professionalism is viewed as a tool to promote anti-discriminatory practice within the clinical landscape [54]. Advocacy is proposed as a valuable competency for trainees to induce changes in institutional professionalism standards [53]. Strategies to centre equity and inclusion include increased justice within professionalism assessment and greater value for diverse populations on clinical teams [65]. Patient facing strategies outlined by studies within this theme include greater humility for structural inequities faced by marginalized populations and attention to positionality in patient care (Fig. 2) [65].

## Discussion

The aim of this scoping review was to characterize existing literature surrounding EDI, cultural humility, and advocacy principles in professionalism education, assessment, or experiences for healthcare trainees and practitioners. EDI and professionalism are explored in three predominant modalities. EDI-interventions such as educational courses improve professionalism outcomes among learners (Fig. 2) [19, 22, 24]. In the present studies, rural and foreign exchange programs are intended to produce improvements in cultural humility. Previous research supports the beneficial outcomes of short-term overseas programs in cultural humility [65, 67]. The present studies extend this finding by demonstrating concurrent improvements in professionalism [32, 34, 38]. Furthermore, there is a direct and positive correlation between professional and cultural competencies measured among practitioners [50]. These findings further support an interconnected model of professionalism and cultural humility.

Interventions promoting advocacy as a domain of professionalism were more limited in the literature [20]. While there is a strong agreement for advocacy as a professional responsibility, the scope of the healthcare practitioner as an advocate remains unclear and may contribute to the lack of organized interventions in this area [68–71]. Nevertheless, Peluso and colleagues (2013) discuss the success of a four-week “advocacy and activism” module as a component of medical students’ professionalism education. Advocacy is frequently cited within the scope of professional competencies of healthcare trainees [72, 73]. Therefore, structured assessment of advocacy education in professionalism remains an area requiring further investigation.

Development and revision of professionalism frameworks rely on values expressed by trainees and practitioners [73, 74]. Studies in the first and second domains of our findings cumulatively express an inherent relationship between EDI and professionalism. Interventions to promote EDI competencies, whether it be through educational courses or clinical placements, foster professionalism amongst learners. Healthcare trainees and professionals conceptualize EDI competencies as core values of professionalism in contrast to existing frameworks that lack EDI domains [45, 75]. Conversely, healthcare practitioners with formed professional identities tend to demonstrate greater awareness for EDI [39]. An application of social learning theory translated from engineering profession consolidates this bidirectional relationship in which successful professional identity formation is necessary to acquire values of EDI [76]. These findings suggest that EDI and professionalism are interrelated domains rather than mutually exclusive competencies.

Commentaries around this topic in the literature signify the disparity faced by individuals from equity-deserving groups due to the lack of EDI integration in current professionalism standards. Narratives from equity deserving groups recall experiences of suppressing their cultural, racial, sexual, and other personal identities to conform with definitions of professionalism. Expression of personal identity that doesn’t align with definitions of professionalism is viewed as unprofessional, hinding individuals from equity deserving groups from attaining positions of leadership [62]. The coin model of privilege and critical allyship, as described by Nixon, conceptualizes each system of inequity as a coin. These coins provide unearned power to certain individuals based on their relationship to the system of inequity. It elegantly highlights the disparity in power to reform systems of inequity disproportionately given to populations of historic domination [77]. In our context, the coin signifies existing standards of professionalism by which health professionals are trained and held accountable. Current standards are viewed as exclusive to professionals from equity-deserving groups, rendering their placement on their bottom of the professionalism “coin” and resulting in an unearned disadvantage. The path towards critical allyship requires efforts from those in positions of privilege to reshape these frameworks, incorporating greater awareness of domains of EDI as core competencies of the health professional. Greater emphasis on the values of EDI in the professionalism standards will help bridge health disparities, reflected in improved patient outcomes and higher quality care [78, 79]. Current challenges to enacting these changes stems from a performative attitude towards professionalism. Professionalism, if reduced to a checklist of behaviours, fails to capture the commitment to social justice required to institute change [63]. Attitudes and beliefs of professionalism assessment also remains a challenge, as trainees are more hesitant to engage in advocacy if their institutions are viewed as hostile towards advocacy efforts [53]. Engaging trainees in the design of professionalism education may prove to be instrumental in instigating change [53].

### Limitations of the literature

Most studies assessed changes in professionalism or measures of EDI with self-reported techniques such as questionnaires, written reflections, or interviews. A few studies use validated tools for assessment of professional development [22] while most rely on the interpretation of student feedback or internally constructed questionnaires [25, 26]. Over the last three decades, an increasing number of validated professionalism tools have been developed based on existing frameworks of professionalism [79, 80]. Professionalism assessment inventories are available across medicine, nursing, and pharmacy and demonstrate high reliability and content validity compared to self-report measures [80–82]. However these tools remain scarce, in-part due to unclear definitions of professionalism, and thus self-report measures are favoured [83]. Increased use of these tools would support more valid assessments of professionalism in the context of EDI-associated interventions. Studies present in this review engaged solely health trainees and professionals with self-assessment of professionalism. Perspectives from patients are a valuable, and often underutilized, source for gauging professionalism in clinical settings [75]. Studies investigating patient perception of professionalism in the context of EDI training may serve as a valuable measure for the integration of these concepts.

### Limitations of our study

The literature search was developed according to the starting framework of the professionalism-in-practice (PIP) developed at McMaster University [3]. The framework provides guidance towards query terms related to domains of EDI. Therefore, the conceptualization of the present literature is within the context of the applied framework. Other frameworks may have informed a different strategy to investigate literature on professionalism [73, 84]. Secondly, our study aims to promote more equity-oriented language according to the AAMC guide to language promoting health equity [11]. In this context, we opted to use terms such as “cultural humility” in place of “cultural competency”. However, we acknowledge that those terms are not interchangeable in all contexts [85].

### Conclusion and future directions

To our knowledge, our study is the first to review literature around EDI and professionalism in the healthcare setting. We demonstrate that a significant body of research supports the integration of domains of EDI into professionalism education and assessment across interdisciplinary programs. Interventions aimed to improve measures of EDI concordantly improved measures of professionalism. Domains of EDI, cultural humility, and advocacy correlate with professionalism noted both by self-reported measures and quantitative surveys of health professionals. Current models of professionalism are viewed as non-inclusive to practitioners of equity-deserving groups. These juxtaposing findings suggest an increasing need for the revision of definitions of professionalism to better address competencies of EDI across healthcare disciplines.

We expect this study to drive future research and serve as a support for the development and revision of professionalism frameworks with domains of EDI, cultural humility, and advocacy. The Michael G. DeGroote school of Medicine from which our work is based recently revised its internal model of professionalism to include a domain of EDI [3]. This revision aims to further address the disparities faced by patients and practitioners of equity-deserving backgrounds. We recommend revisions in professionalism frameworks to serve as a foundation for deeper implementation of EDI in existing health professions training through various modalities including educational opportunities, tools, and mentorship programs. The literature reviewed in this study support the use of interprofessional educational courses. Alternatively electives that increase exposure to equity deserving groups is a feasible form of EDI training for professionalism development. EDI is also increasingly being incorporated into healthcare simulation with recent development of tools for trainees to reflect on simulation training from an EDI perspective [85, 86, 87]. In addition to educational courses and clinical placements discussed in this review, individualized support towards professional identity formation of trainees from equity deserving groups is in emergently recognized aspect of professionalism education [88]. The University of Toronto’s diversity mentorship initiative have successfully aimed to support professionalism among students from equity-deserving groups, demonstrating the effectiveness of structured mentorship in fostering professional growth within these populations [88]. Incorporation of EDI into professionalism education for health professionals is a longitudinal endeavour starting at revision of existing frameworks and definitions of professionalism., We hope our work drives the evidence-based design of professionalism frameworks guiding health professional education and standards of assessment.

## Electronic supplementary material

Below is the link to the electronic supplementary material.

Supplementary Material 1

## Data Availability

The authors confirm that all relevant data, including the search strategy, databases used, and the papers used in data extraction and analysis are included in the article, or can be found in the supplementary files.

## Abbreviations

EDI: Equity, Diversity and Inclusion
MERSQI: Medical Education Research Study Quality Instrument
MGDSM: Michael G. DeGroote School of Medicine
NGT: Nominal Group Technique
NTUCM: National Taiwan University College of Medicine
PIP: Professionalism in Practice
PRISMA-ScR: Preferred Reporting Items for Systematic Reviews and Meta-Analyses extension for Scoping Reviews
