# Supplementary material for: Incorporating equity, diversity and inclusion (EDI) into the education and assessment of professionalism for healthcare professionals and trainees: a scoping review
Source: BMC Med Educ. 2024 Sep 11;24:991. doi: 10.1186/s12909-024-05981-3 (PMC11391843; doi:10.1186/s12909-024-05981-3)
Supplement: Supplementary file 1 — Supplementary Material 1 [file 12909_2024_5981_MOESM1_ESM.docx]

**Additional Files:**

File name: Additional File 1.docx

Appendix S1**:** Search strategy

Summary of search strategy used in the scoping review. Reviewed databases include Medline,

EMBASE, and PsycInfo.

Appendix S2: Table 1: Characteristics of included studies theme 1; Table 2: Characteristics of included studies in theme 2; Table 3: Characteristics of included studies in theme 3

Summary of 48 studies extracted in this review organized by theme. Outcomes include study characteristics, methodology, population of interest, and professionalism outcomes.

Appendix S3: Table 1: MERSQI quality appraisal scores of quantitative studies; Table 2: Cote quality appraisal scores of qualitative studies

Quality and risk of bias assessment for quantitative (MERSQI tool) and qualitative (Cote et al. tool) studies reviewed in this article.

**Appendix S1**

Search strategy

| **OVID Medline Epub Ahead of Print, In-Process & Other Non-Indexed Citations, Ovid MEDLINE(R) Daily and Ovid MEDLINE(R) 1946 to March, 7 2023**  1 health personnel/ or allied health personnel/ or anatomists/ or anesthetists/ or  audiologists/ or caregivers/ or "coroners and medical examiners"/ or dental staff/ or dentists/ or  doulas/ or emergency medical dispatcher/ or epidemiologists/ or faculty, dental/ or faculty,  medical/ or faculty, nursing/ or health educators/ or health facility administrators/ or infection  control practitioners/ or medical chaperones/ or medical laboratory personnel/ or medical staff/  or nurses/ or nursing staff/ or nutritionists/ or occupational therapists/ or optometrists/ or  personnel, hospital/ or pharmacists/ or physical therapists/ or physician executives/ or  physicians/ or psychotherapists/ or traditional medicine practitioners/ 383996  2 exp Students, Health Occupations/ 85729  3 (Physician* or Physician assistant* or Residen* or Medical student* or Nurse or Midwife  or Midwifery or Physiotherapist* or Physical therapist* Occupational therapist or Speech  Language Pathologist or Pharmacist or Social Worker* or Psychiatrist* or Therapist* or  Dietician*).mp. [mp=title, book title, abstract, original title, name of substance word, subject  heading word, floating sub-heading word, keyword heading word, organism supplementary  concept word, protocol supplementary concept word, rare disease supplementary concept word,  unique identifier, synonyms, population supplementary concept word, anatomy supplementary  concept word] 1273454  4 exp ethics, clinical/ or exp professionalism/ 66291  5 professional competence/ or clinical competence/ 128571  6 (professional responsibility or professionalism responsibility).mp. [mp=title, book title,  abstract, original title, name of substance word, subject heading word, floating sub-heading  word, keyword heading word, organism supplementary concept word, protocol supplementary  concept word, rare disease supplementary concept word, unique identifier, synonyms,  population supplementary concept word, anatomy supplementary concept word] 1216  7 professional identit*.mp. 3073  8 professional integrity.mp. 252  9 professionalism.mp. 10276  10 (professional behavio?r or professionalism behavio?r).mp. [mp=title, book title, abstract,  original title, name of substance word, subject heading word, floating sub-heading word,  keyword heading word, organism supplementary concept word, protocol supplementary concept  word, rare disease supplementary concept word, unique identifier, synonyms, population  supplementary concept word, anatomy supplementary concept word] 1046  11 (Professional practice* or professionalism practice).mp. [mp=title, book title, abstract,  original title, name of substance word, subject heading word, floating sub-heading word,  keyword heading word, organism supplementary concept word, protocol supplementary concept  word, rare disease supplementary concept word, unique identifier, synonyms, population  supplementary concept word, anatomy supplementary concept word] 26450  12 professional expectation*.mp. 187  13 (professional development or professionalism development).mp. [mp=title, book title,  abstract, original title, name of substance word, subject heading word, floating sub-heading  word, keyword heading word, organism supplementary concept word, protocol supplementary  concept word, rare disease supplementary concept word, unique identifier, synonyms,  population supplementary concept word, anatomy supplementary concept word] 13194  14 Cultural Competency/ or cultural humility.mp. 6809  15 race-consciousness.mp. 20  16 cultural safety.mp. 667  17 Culturally Competent Care/ 2074  18 EDI.mp. 3575  19 diversity, equity, inclusion/ 47  20 health inequities/ 298  21 Health Equity/ 3348  22 Healthcare Disparities/ 21820  23 health advocacy.mp. 722  24 or/1-3 1520992  25 or/4-13234574  26 or/14-23 37636  27 24 and 25 and 26 776 |
| --- |
| **Embase <1974 to 2023 March 07>**  1 exp health care personnel/ 1938320  2 exp health student/ or exp medical student/ 137493  3 (Physician* or Physician assistant* or Residen* or Medical student* or Nurse or Midwife  or Midwifery or Physiotherapist* or Physical therapist* Occupational therapist or Speech  Language Pathologist or Pharmacist or Social Worker* or Psychiatrist* or Therapist* or  Dietician*).mp.1809447  4 professional practice/ or professionalism/ or professional competence/ 98733  5 professionalism/ 11088  6 professional competence/ 33931  7 (professional responsibility or professionalism responsibility).mp. 1552  8 professional identit*.mp. 3498  9 professional integrity.mp. 308  10 professionalism.mp. 17177  11 (professional behavio?r or professionalism behavio?r).mp. 1237  12 (Professional practice* or professionalism practice).mp. 63094  13 professional expectation*.mp. 236  14 (professional development or professionalism development).mp. 22740  15 exp cultural competence/ 7859  16 cultural humility.mp. 602  17 (race-consciousness or race conscious*).mp. [mp=title, abstract, heading word, drug  trade name, original title, device manufacturer, drug manufacturer, device trade name, keyword  heading word, floating subheading word, candidate term word] 87  18 transcultural care/ 5023  19 exp health equity/ 9156  20 exp health disparity/ 32370  21 health advocacy.mp. 875  22 EDI.mp. 4900  23 or/1-3 2757824  24 or/4-14132020  25 or/15-22 57509  26 23 and 24 and 25 768 |
| **APA PsycInfo <1806 to March 7 2023>**  1 exp health personnel/ 186382  2 health personnel/ or allied health personnel/ or anatomists/ or anesthetists/ or  audiologists/ or caregivers/ or "coroners and medical examiners"/ or dental staff/ or dentists/ or  doulas/ or emergency medical dispatcher/ or epidemiologists/ or faculty, dental/ or faculty,  medical/ or faculty, nursing/ or health educators/ or health facility administrators/ or infection  control practitioners/ or medical chaperones/ or medical laboratory personnel/ or medical staff/  or nurses/ or nursing staff/ or nutritionists/ or occupational therapists/ or optometrists/ or  personnel, hospital/ or pharmacists/ or physical therapists/ or physician executives/ or  physicians/ or psychotherapists/ or traditional medicine practitioners/ 122746  3 exp medical students/ 14674  4 (Physician* or Physician assistant* or Residen* or Medical student* or Nurse or Midwife  or Midwifery or Physiotherapist* or Physical therapist* Occupational therapist or Speech  Language Pathologist or Pharmacist or Social Worker* or Psychiatrist* or Therapist* or  Dietician*).mp. [mp=title, abstract, heading word, table of contents, key concepts, original title,  tests & measures, mesh word] 387273  5 exp professionalism/ 4168  6 exp professional standards/ 9644  7 exp professional competence/ 13510  8 (professional responsibility or professionalism responsibility).mp. [mp=title, abstract,  heading word, table of contents, key concepts, original title, tests & measures, mesh word] 702  9 (professional identit* or professional integrity or professionalism).mp. 14419  10 (professional behavio?r or professionalism behavio?r).mp. [mp=title, abstract, heading  word, table of contents, key concepts, original title, tests & measures, mesh word] 680  11 (Professional practice* or professionalism practice).mp. [mp=title, abstract, heading  word, table of contents, key concepts, original title, tests & measures, mesh word] 8483  12 professional expectation*.mp. 165  13 (professional development or professionalism development).mp. [mp=title, abstract,  heading word, table of contents, key concepts, original title, tests & measures, mesh word] 35869  14 exp professional development/ 71683  15 exp professional identity/ 4745  16 1 or 2 or 3 or 4 485110  17 5 or 6 or 7 or 8 or 9 or 10 or 11 or 12 or 13 or 14 or 15 99185  18 exp cultural sensitivity/ 8152  19 (cultural humility or cultural Competency).mp. 4071  20 race-consciousness.mp. 96  21 cultural safety.mp. 259  22 exp health personnel attitudes/ 26837  23 exp cross cultural treatment/ 4280  24 EDI.mp. 1268  25 exp diversity/ 16480  26 exp equity/ 24070  27 exp inclusion/ 10736  28 exp health disparities/ 10673  29 health inequities.mp. 1113  30 health equity.mp. 1748  31 health advocacy.mp. 375  32 18 or 19 or 20 or 21 or 22 or 23 or 24 or 25 or 26 or 27 or 28 or 29 or 30 or 31 92330  33 16 and 17 and 32 2759 |

**Appendix S2**

Table 1: Characteristics of included studies in theme 1

| Author, Year | Country of study | Study design | Methodology | Sample size (N) | Population description | Program description | Type of outcome measure | Professionalism outcomes |
| --- | --- | --- | --- | --- | --- | --- | --- | --- |
| Midik, 2016 [19] | Turkey | After attending this elective, medical students completed questionnaires that underwent descriptive qualitative and quantitative analyses. | Mixed-method | 60 (3 groups x 20 participants in each) | Medical students | A student-centered problem-based elective program for medical students consisting of lectures, panel discussions, small reflection groups and a training program to improve students' skills and knowledge on communicating with hard of hearing people and Turkish sign language competency. | Self-report likert scale and reflective portfolios | Medical students reported that the program contributed to their professionalism. |
| Midik, 2020 [20] | Turkey | Qualitative analysis using pre- and post-sessions and student feedback about the "Roles of the Doctor" program. | Qualitative | 47 | Medical students | The elective program "Roles of the Doctor" contained a curriculum that focused on health advocacy, created using various approaches such as experimental, inquiry-based, and community-based learning. This program aims to help medical students recognize and develop professional identity. | Written reflections | 45 out of 47 students were satisfied at the end of the elective program and stated that the elective was beneficial for them in terms of raising their awareness of their professional identities. They developed competencies in communication, scholarship, health advocacy and high skill level.  The elective program was beneficial in raising awareness of professional identities. Students developed competencies in communication, scholarship, and health advocacy |
| Kacan, 2020 [21] | Turkey | A post-test quasi-experimental study with an experimental and control group was conducted to evaluate professional values, cultural sensitivity and empathy of 2nd-year nursing students enrolled in a transcultural nursing course. | Quantitative | 125 (experimental group = 65, control group = 60) | Nursing students | In this program, topics such as health and culture, nursing and culture, transcultural nursing and historical development, and cultural sensitivity were taught in theoretical classes. | Nurses professional values scale revised form, Empathic skill scale-B form (ESS-B), Intercultural sensitivity scale (ISS), Cultural intelligence scale (CIS) | The transcultural nursing program helps improve students' cultural sensitivity and cultural intelligence, professional values, and empathy. |
| Lee, 2019 [22] | USA | Qualitative study based on the thematic analysis of student reflections. | Qualitative | 40 | Medical students | The two Native Hawaiian Center of Excellence community health electives, named Kuaola and Na ʻOpio o Nānākuli, require first year medical students to mentor high school and/or intermediate students. At midyear, students participating were asked to complete a reflection essay describing areas of professionalism that were enhanced or developed. | Written reflection | The service learning events and adolescent mentoring enhanced first year medical students’ understanding of cultural humility, native Hawaiian rural student life, professionalism, humanism in medicine, responsibility, empathy, communication, compassion, and the need for self-reflection. |
| Fleming, 2020 [23] | Australia | Qualitative study with semi-structured interviews after a series of workshops and yarning circles which is a form of traditional indigenous storytelling. Results were analyzed by thematic analysis. | Qualitative | 8 | Midwifery academics teaching on a bachelor of Midwifery program at a metropolitan Australian university | Two half-day workshops and six yarning circles over 12 weeks. The yarning circles focused on topics of respect, communication, safety, advocacy, and leadership. Participants also completed reflective journals. | Semi-structured interviews | The program was effective at promoting professional development with regards to cultural safety among indigenous peoples . It encouraged greater personal and professional reflection. |
| Rosen,2017 [24] | USA | Students completed three open-ended questions as an evaluation of the assignment. Answers were coded into categories for qualitative data analysis. | Qualitative | 47 | Social work graduate students | This program provides frameworks for understanding the influence of race/ethnicity, class, gender, sexuality, age, and ability on individuals. It also contains concepts of oppression, privilege, and cultural humility, followed by a presentation by students. | Written reflection | Students described the course helped develop their professional identity. It also promotes reflection of their personal identity in the context of race, class, gender, age, ability which facilitated cultural humility. |
| Awaad, 2015 [25] | USA | Psychiatry residents attended a religion and spirituality training course, and completed a modified version of the Course Impact Questionnaire (a 20-item Likert scale to assess residents' spiritual & professional attributes), as well as written comments (feedback for the course). | Quantitative | 20 | Psychiatry residents | A six-session course with didactic lectures and process-oriented discussions led by teams of 2 faculty members. Discussions were about addressing spirituality and religion in patient care and interactions. | Self-report Course Impact questionnaire | Psychiatry residents experienced a significant improvement in professional practice and attitude scores after attending the religion and spirituality program. |
| Brown, 2008 [26] | USA | Students were given two separate pre and post-course surveys to assess ability to work in interprofessional teams and perceived cultural competence. The Student Attitude Questionnaire assesses interprofessional education and the Inventory for Assessing the Process of Cultural Competence Among Health Care Professionals Revised was used to assess cultural competence. | Quantitative | 73 | Graduate and undergraduate students in nursing, pharmacy, social work, and other allied health programs. | A cased-based didactic course to develop interprofessional team behaviours and foster the development of cultural competency within health professions students. Students also participated in group discussions, reflections, and peer assessments. | Self-report Student Attitude Questionnaire and Inventory for Assessing the Process of Cultural Competence Among Health Care Professionals- Revised (IAPCC-R) | The course improved both cultural awareness and professionalism through increased understanding of interprofessional interactions. Older students and caucasian students had greater changes in pre to post test scores. |
| Daly, 2013 [27] | Australia | Qualitative study with semi-structured interviews exploring the experiences of participants during a rural clinical placement. | Qualitative | 42 | Medical students, general practitioners, and hospital clinicians | 6-12 month rural placement. Students rotate between general practice, hospital, remote, and academic settings to improve preparedness for practice. | Semi-structured interviews | Students reported growth in professional identity and development of self-efficacy. It provided opportunities to build resilience and characterize boundaries in knowledge and skills. In parallel, the placement improved cultural awareness with greater understanding of cultural determinants of health and resource issues in rural settings. |
| Glaser, 2019 [28] | USA | Mixed-method study involving analysis from electronic surveys and semi-structured interviews about students' clerkship experience. | Mixed-method | 103 | Medical students | The University of California, San Francisco School of Medicine offers diverse clerkship opportunities at highly-rated institutions. | Self-report survey and semi-structured interviews | Clinical clerkship experiences exposed students to health care disparities, impacting their professional development both positively and negatively. While some students became cynical and developed a physician-centered view with emotional burden, others expressed a strong commitment to combat disparities, rejecting acculturation pressure, improving advocacy skills, and increasing motivation for equitable care. |
| Jansen, 2021 [29] | Cypress, USA, Turkey, Kenya, Finland, UK, Ireland, Norway, Thailand, Tanzania, Denmark | Qualitative synthesis of written reflections from nursing students before, during, and after the stay. | Qualitative | 23 | Nursing students | Nursing students engage short-term (3-4 weeks) or long-term (10-12 weeks) international clinical placements as part of their nursing education program. All participating countries are established partners within the Erasmus bilateral agreements. | Written reflections | Professional growth was experienced as an improvement of nursing skills, including in decision-making, cultural competency, professional judgment, communication, and observation. The professionalism they developed during the ICP gave the participants a direction for their future job as qualified nurses. |
| Knecht, 2020 [30] | USA | A mixed-method study involving collecting quantitative data from surveys, and qualitative, written response data before and after the exchange program. The pretravel survey inquired students' perspectives about the importance of the different aspects of their service-learning program. The post-travel survey inquired about the learning opportunities students were exposed to. | Mixed-method | 43 | Undergraduate and graduate nursing students | Nursing students attended health, social and education programs in Kenya. These programs involved assignments, engaging in community-based and acute-care settings in urban and rural areas. Students collaborated with healthcare and social services providers in tasks such as outpatient clinic visits, and screening. The ultimate goal was to grow students' professional, personal and cultural competencies. | Self-report survey and written reflection | Professional development was one of the themes that emerged from students' pre- and post-travel comments, with specific areas of growth noted (e.g. leadership, critical thinking, teamwork, providing care more sustainably, etc.) |
| Yao, 2016 [31] | USA | Cross sectional study with an electronic survey on attitudes towards the Smile Regan/Stryker fellowship programs. | Quantitative | 165 | Plastic surgeons and plastic surgery residents | 2-week surgical mission in a resource limited, multicultural setting with a variety of impacting socioeconomic factors, followed by a debrief session in a peer and mentor group. | Self-report surveys | The goal of the program was to foster cultural competence through international experiences. 96% of participants agreed that the experience improved professionalism as it allowed fellows to engage with a culturally diverse group of professionals. |
| Zebryk, 2021 [32] | Poland | Mixed-methods study with qualitative and quantitative data collected via a self-report questionnaire. | Mixed-method | 109 | Medical students | This program requires students to study at a foreign institution for 3-12 months. The goals of the program are personal and professional development, experience of other cultures, and comparison of medical education. | Self-report survey | Participants report a major difference in professionalism across different cultures within the patient-physician relationship when compared with their home country. Consequently, participants reported increased professional development after the experience. |
| Koskinen, 2009 [33] | Canada, England, Estonia, Finland and Sweden | Students wrote 'critical incidents' during their international exchange programs, with the written excerpts analyzed, and subsequently categorizing and identifying student feelings and emotions from their experience. | Qualitative | 48 | Nursing students | An 8-16 week trans-Atlantic rural community placement for European and Canadian undergraduate nursing students to improve students' knowledge of social inequities and cultural barriers in healthcare. | Written reflection | One of the learning themes through which students' cultural competence was enhanced was professional awareness. Students’ interpretations of professionalism were challenged through their encounters with personal ethical situations. The exchange program demonstrated an improvement in students' professional awareness, with progressive improvement as students' adjusted better in the host country's culture. |
| Behar-Horenstein, 2015 [34] | USA | Dental students who attended the service learning program completed 12 reflective writing assignments and an interview at the end of the program to broadly inquire about what student's took away from their experience. | Qualitative | 5 | Dental students | A service learning experience for dental students which involved placements in at-risk healthcare settings (e.g. a homeless center, medical organization, and a county health department) prior to the start of dental school. Students also planned activities for preventative health and patient education. | Reflective writing assignments | The service-learning program impacted the students' beliefs about cultural competence, and assisted in professional identity development. |
| Green, 2008 [35] | UK, Sweden | Case study based on semi-structured interviews and documentary analysis. | Qualitative | 32 | Nursing students | International study programmes for nursing students were organized by a school in the UK and a school in Sweden. Placement locations included in Norway, Denmark, Spain, Holland, USA, South Africa and Hong Kong. The duration of the visits for the UK participants was 12 weeks. The duration of the visits for the Swedish participants ranged from five to 20 weeks. | Semi-structured individual and group interviews, and documentary analysis | Students indicated that their professional development encompassed both knowledge and practical skills, leading to a heightened awareness of disparities in healthcare practices and the role of nurses. They also reported increased awareness of diverse cultural values, which fostered tolerance towards others and a deeper understanding of challenges faced by ethnic minority groups. |
| Hande, 2022 [36] | Sweden, India | Semi-structured interviews with qualitative content analysis. Questions explored experiences, personal development, and professional development. | Qualitative | 6 | Physical therapists or teachers in physical therapy | Three month exchange program between Sweden and India physiotherapists or physiotherapy students. | Semi-structured interviews | Participants gained cultural and environmental experiences with improvement in patient communication, taking feedback. There was increased open-mindedness. Swedish participants in particular had increased cultural awareness which directly improved professional development. |
| Lee, 2007 [37] | Taiwan, Macau, Chinese Mainland and Hong Kong | A mixed-method (qualitative and quantitative) analysis of students' perspectives on the exchange program, and their reported personal and professional development using a post-program questionnaire and focus group interviews. | Mixed-method | 64 | Nursing students | A 2-week, nursing summer exchange program between 15 universities in Taiwan, Macau, Hong Kong and Chinese mainland that provided students with a health counselling course (workshops and lectures), as well as community placements. | Self-reported questionnaire and focus group interview | Students reported having a greater awareness of cultural diversity in the context of their professional and personal development. |
| Nishigori, 2009 [38] | Japan, UK | Thematic analysis of semi-structured individual interviews | Qualitative | 21 | Medical students | The Japan Medical Education Foundation (JMEF) annually organizes and funds international electives in the UK for Japanese medical students every year to study clinical medicine. Additionally, British medical students from two schools were placed through the JMEF in clinical clerkships in Japan. | Semi-structured individual interviews | Participation in international electives was associated with several outcomes related to medical professionalism. This includes understanding their commitment to improving access to care, a just distribution of finite resources, and to professional competence. |
| Callister, 2006 [39] | Argentina, Guatemala, Jordan, Ute & Navajo Nations | Van Manen’s method of phenomenological analysis was utilized for data analysis of transcribed audiotapes. | Qualitative | 20 | Recent nursing graduates | To understand the meaning and significance of former nursing students' participation in an international clinical nursing elective, participants were contacted by telephone, email, or mail and were invited to participate in the study. Those willing to participate were sent consent and demographic forms. | Individual interviews | Participants reported that the international clinical nursing elective contributed to their professional development by modeling evidence-based practice, demonstrating holistic nursing practice, and participating in international nursing conferences. They also developed cultural competence, increasing their sensitivity and awareness to other beliefs and cultures. |

Table 2: Characteristics of included studies in theme 2

| Author, Year | Country of study | Study design | Methodology | Sample size (N) | Population description | Program description | Type of outcome measure | Professionalism outcomes |
| --- | --- | --- | --- | --- | --- | --- | --- | --- |
| Chae, 2020 [40] | South Korea | A descriptive, cross-sectional study that determined individual- and systemic-level factors that impact the cultural competency skills of nurses through voluntary responses to surveys. Individual variables, demographics, professionalism, systemic-level factors were some of the key measured factors. | Qualitative | 401 | Nurses | This study recruited nurses that work directly with culturally-diverse patient populations, and researched individual- and organizational-level factors that influence cultural competency. | Cultural Competence Scale for Nurses-Short Form (CCSN-SF), Professionalism Inventory Scale, Practice Environment Scale of Nursing Work Index  (PES-NWI), Organisational Cultural Competence Measure for Human Service  Agencies | Professionalism was a significant predictor of nurses' cultural competency. |
| Adams, 2011 [41] | Australia, USA, Germany, Canada, New Zealand, UK, Israel | An integrative literature review on papers that involve primary collection of data on knowledge and attitude on or professional practice and referrals to complementary and alternative medicine by midwives, obstetricians, and other maternity professionals. | Qualitative | N/A | N/A | N/A | Literature search | Understandings and perceptions of complementary and alternative medicine (CAM) seem to be associated with the broader concepts of professionalism and professional identity. Many midwives support that having an understanding of CAM in their patients' lives is them 'meeting the professional needs of their patients', allowing for holistic care. |
| Fleming, 2019 [42] | Australia, New Zealand, Canada and USA | This integrative literature review selected for original research that evaluated cultural safety programs. Additionally, the participants of said programs had to be academics teaching in a midwifery program. | Qualitative | N/A | N/A | Cultural safety programs taught by academics teaching in a midwifery program. | Literature search | Cultural safety is needed to be incorporated into professional development curricula for midwifery academics. In some of the papers described in the literature review, cultural safety helped guide professional development interventions. Participants of professional development courses report improvements in cultural knowledge. |
| Teasley, 2005 [43] | USA | Exploratory research focusing on the analysis of self-report survey research methods and quantitative data. | Quantitative | 247 | Social workers | N/A | Cultural  Competence Self-Assessment Questionnaire | Respondents reported professional development to be a greater factor than formal education in the determination of their levels of cultural competence. The use of professional literature and workshops were identified as significant factors for the development of culturally competent urban school-based practitioners. |
| Ho, 2017 [44] | Taiwan | Qualitative thematic analysis of focus group interviews. | Qualitative | 64 | Medical students | Participants shared their understanding of professionalism in medical school. They also discussed experiences of professional dilemmas and ethical dilemmas. | Narrative interview | Half of all reported professionalism dilemmas are related to cultural and intercultural conflict. This was paired with a low advocacy and low inquiry approach to professionalism dilemmas suggesting avoidance. |
| Ly, 2013 [45] | USA | Systematic literature search of MEDLINE, EBSCOhost, Web of Science, and Google Scholar Databases. | Qualitative | N/A | N/A | Five programs were reviewed which included lectures, objective structured clinical examinations, and written exercises and evaluations. | Literature search | The majority of programs such as the surgical professionalism in clinical education (SPICE) program addressed cultural competency during assessment of professionalism. Residents had improved professionalism and communication skills. The curriculum implemented at the Department of Surgery at the University of Hawaii showed no improvement, potentially due to negative attitudes towards cultural training. |
| Crenshaw, 2011 [46] | USA | Qualitative cluster analysis. Nominal group technique used to generate concepts related to a cultural competence curriculum program. These concepts were sorted by educators and researchers, and analyzed into clusters. | Qualitative | 30 | Medical students, internal medicine residents, community physicians and researchers. | Internet-based, cultural competence curriculum program for physicians and physicians-in-training to reduce health disparities in cardiovascular health. | Structured small group meeting | Four major clusters were identified, one of which was the provider and health care. Within this cluster, addressing issues in professionalism was identified as an important component to a cultural competency curriculum predominantly by medical students. |
| Minicuci, 2020 [47] | UK | Quantitative study analyzing physician questionnaires. | Quantitative | 374 | Doctors attending Continuing Medical Education (CME) courses | As organized by the Medical Association of the Province of Padua, doctors attending Continuing Medical Education (CME) courses were administered an anonymous self-administered questionnaire during a five-month period to assess their perception of professional values. | Self-reported questionnaire | Participants rated the importance of professional values in the following order (from most to least important): competence, responsibility, integrity, commitment, advocacy, confidentiality, spirit of inquiry and lastly, compassion. |
| Matthews, 2018 [48] | South Africa | Exploratory cross-sectional analytical study of quantitative survey data from a self-administered questionnaire. The survey addressed themes of perceptions of cultural competence in relation to the curriculum. | Quantitative | 142 | Medical students | The program provides students with communication skills courses, intercultural communication training, and working directly with patients in urban and rural settings. | Self-reported questionnaire | Most students agreed that more professional development was required to provide services and support culturally and linguistically diverse groups. Participants also agreed that the experience improved their awareness of health care disparities and ability to communicate with patients from different cultural backgrounds. |
| Verdon, 2020 [49] | Australia | Participants completed anonymous evaluation surveys before and after the workshop regarding their demographic data, practice experience, language skills, their confidence in working with marginalized populations, their challenges, as well as how participants felt about working with these populations after completing the program. Two participants were followed up via an interview a year following the workshop to investigate any long-term impacts of the workshop. | Mixed-method | 70 | Multidisciplinary healthcare professionals (e.g. social workers, speech pathologists, nurses) | Multidisciplinary professionals attended a 3-hour professional development workshop based on the Principles of Culturally Competent Practice (PCCP) framework to help create sustainable changes in their cultural competency skills. | Pre- and post- workshop questionnaires, and interviews | Participants reported that their motivation for undertaking professional development opportunities were to gain a deeper insight of linguistic and cultural diversity, as well as to help benefit the children and families they worked with. Also, professional development workshops foster a reflective environment for professionals to think about their own practice and existing services to create culturally-safe spaces for their patients. |
| Hamdan Alshammari, 2022 [50] | Saudi Arabia | A correlational, cross sectional design using the structural equation model (SEM). | Mixed-method | 587 | Tertiary care nurses | N/A | Self-reported questionnaires | The study found that different dimensions of professional competency are interrelated. Value-based nursing care, care pedagogics, and medical and technical care have a significant impact on nurses' cultural competency. Moreover, the dimension of leadership, development and organization of nursing care positively affected all other dimensions of professional competency, particularly those involving patient-related nursing and organization and development of nursing care. |
| Howells, 2016 [51] | Australia | Mixed methods design with pre and post-placement surveys, reflections and focus groups. Speech-language pathology students completed a survey at the beginning and end of their clinical placements to explore the impact of clinical placements on students' perceptions and knowledge on working with culturally-diverse populations. | Mixed-method | 60 | Master of Speech Pathology students | No particular cultural competency program was implemented. Students' experiences were simply evaluated after the completion of their general clinical placements in which students occasionally worked with culturally-diverse groups. | Initial cultural awareness survey, pre- and post-placement written reflection, post-placement focus group | Content analysis of focus groups and written reflection data of students revealed professional and cultural competency development. |

Table 3: Characteristics of included studies in theme 3

| Author, Year | Country of study | Study design | Methodology | Sample size (N) | Population description | Program description | Type of outcome measure | Professionalism outcomes |
| --- | --- | --- | --- | --- | --- | --- | --- | --- |
| Alexis, 2020 [52] | USA | Mixed-methods analysis of survey data and qualitative narrative data of faculty, trainees, staff and students affiliated with health systems and health professional/graduate schools at the University of Pennsylvania on their perceptions of professionalism | Mixed-method | 3506 | Healthcare trainees & teaching staff | N/A | Survey | Many trainees and staff who self-identified as members of marginalized groups expressed higher infringement on their professional boundaries in work and learning (e.g. racism, homophobia, etc.), and that professionalism standards were applied disproportionately to certain groups over others. Some narrators expressed not being welcomed and experiencing tension between inclusion and assimilation. The study finds it is most valuable to examine professionalism through the lens of inclusion. |
| Maristany, 2023 [53] | USA | Qualitative analysis of interview responses from 4th-year medical students and senior residents at different institutions about perceptions and experiences with professionalism | Qualitative | 49 | Fourth-year medical students at three diverse medical schools across the USA, as well as senioras. Moreover, senior residents in family medicine, internal medicine, obstetrics-gynecology, pediatrics, general surgery and neurosurgery. |  | Semi-structured interviews | Findings show that professionalism can act as an oppressive force that reinforces white, male norms and an empowering tool that can extend agency to marginalized communities, affecting trainees' comfort, mental health and professional evaluations |
| Leyerzapf, 2014 [54] | Netherlands | A thematic and integral content analysis of physician and resident experiences of performance appraisal | Qualitative | 27 | Physicians and residents from cultural minorities |  | Semi-structured interviews and focus groups | Social norms on professionalism deem criteria for performance appraisal. These social norms are stereotyped against individuals from cultural minorities. |
| Osseo-Asare, 2018 [55] | USA | Constant comparative analysis of semi-structured interviews to charactierize how Black, Hispanic, and Native American resident physicians experience race/ethnicity in the workplace. | Qualitative | 27 | Residents who self-identified as black, Hispanic, and/or Native American |  | Semi-structured interviews | Residents from minority backgrounds had challenges negotiating professional and personal identities. Expression of personal identity came with the implication that there was a lack of professionalism. There is also a scarcity in professional mentorship available. |
| Kristoffersson, 2022 [56] | Sweden | Interviews on topics of medical school experiences, interactions with colleagues and patients, and other situations influenced by their background | Qualitative | 15 | Medical students who identified as having ethnic or cultural minority backgrounds | N/A | Semi-structured interviews d | Students find that current definitions of professionalism implicitly assess students' capacity to assimilate with the norms of the white majority. Furthermore, addressing issues of racism is viewed as a lack of professionalism |
| Bullock, 2020 [57] | USA | N/AExplanatory analysis of survey data to characterize the prevalance of racial and ethnic sterotype threat experienced by fourth-year medical students | Qualitative | 184 | Fourth-year medical students | N/A | Quantitative Stereotype Vulnerability Scale followed by semi-structured interviews | Students described the notion that professionalism correlated with less ethnic expression and greater conformity to a "White" professional standard. |
| Chandratilake, 2021 [58] | UK, Greece, Portugal, Germany, Denmark, South Africa, Nigeria, Sudan, Sri Lanka, India, Saudi Arabia, Japan, Australia, New Zealand, Canada, USA, Chile | Medical practitioners across multiple countries were surveyed regarding the 'essentialness' of forty-six professionalism attributes the authors had identified from a literature search | Mixed-method | 584 | International medical educators enrolled in the Postgraduate Certificate, Diploma and Masters courses at the Centre for Medical Education, University of Dundee, UK. | N/A | Cross-sectional survey | The study examined the perception of medical professional attributes across geographic locations. Findings confirmed 29 attributes that are universally accepted, but 11 attributes that varied by region, suggesting that perceptions may be impacted by social, economic and cultural backgrounds |
| Cerdeña, 2022 [59] | Not reported | N/A | Commentary | N/A | N/A | N/A | N/A | The authors call for redefining professionalism to prioritize patient welfare, autonomy and social justice and to accommodate for the diverse life experiences and perspectives among medical trainees and practitioners |
| Allitt, 2022 [60] | Not reported | N/A | Commentary | N/A | N/A | N/A | N/A | The authors advocate for a humanities-led approach to teach professionalism, engaging with topics like history, literature, and arts. This can enable medical students to critique the rigid professional norms to foster inclusivity |
| Rosenberg, 2021 [61] | USA | N/A | Commentary | N/A | N/A | N/A | N/A | Commentary on the necessity of equity and inclusion practices to be included in our definition of professionalism |
| AbdelHameid, 2020 [62] | USA | N/A | Commentary | N/A | N/A | N/A | N/A | Narrative on the challenges of professionalism for Black women in Medicine |
| Frye, 2020 [63] | USA | N/A | Commentary | N/A | N/A | N/A | N/A | The authors argue that operationalizing professionalism as a checklist of behaviours may reinforce dominant white norms, making it a tool for social control rather than a mechanism for genuine change. |
| Goddard, 2022 [64] | Australia, China, Germany, Japan, Saudi Arabia, South America, South Africa, Netherlands, UK, USA, | N/A | Commentary | N/A | N/A | N/A | N/A | The authors argue that the definition of professionalism is not universally agreed upon across different cultures and nations. It suggests that two-way discourse between the "profession" and "public" must take place to align professionalism with evolving societal needs |
| McKimm, 2015 [65] | UK | N/A | Commentary | N/A | N/A | N/A | N/A | The definition of “professionalism” changes with societal, cultural and individual norms. The authors suggest that doctors view it as a belief system embedded within an evolving professional identity to arrive at cultural competency. |
| Wynia, 2014 [66] | USA | N/A | Commentary | N/A | N/A | N/A | N/A | Redefines professionalism as a "motivational force or belief system that leads clinicians to come together, in groups and often across occupational divides, to create and keep shared promises". |

**Appendix S3**

Table 1: MERSQI quality appraisal scores of quantitative studies

| Lead author | MERSQI Score |
| --- | --- |
| Midik [19] | 12 |
| Kacan [21] | 12 |
| Awaad [25] | 11 |
| Brown [26] | 8.5 |
| Glaser [28] | 10.5 |
| Knecht [30] | 9.5 |
| Yao [31] | 7 |
| Zebryk [32] | 7 |
| Lee [37] | 12 |
| Teasley [43] | 12.5 |
| Minicuci [47] | 11 |
| Matthews [48] | 8 |
| Verdon [49] | 10 |
| Hamdan Alshammari [50] | 13 |
| Howells [51] | 14.5 |
| Alexis [52] | 11 |
| Chandratilake [58] | 10 |

Table 2: Cote quality appraisal scores of qualitative studies

| Lead author | Cote Score |
| --- | --- |
| Midik [20] | 11 |
| Lee [22] | 9 |
| Fleming [23] | 12 |
| Rosen [24] | 12 |
| Daly [27] | 11 |
| Jansen [29] | 12 |
| Koskinen [33] | 11 |
| Behar-Horenstein [34] | 12 |
| Green [35] | 11 |
| Hande [36] | 12 |
| Nishigori [38] | 11 |
| Callister [39] | 11 |
| Chae [40] | 11 |
| Adams [41] | 11 |
| Fleming [42] | 8 |
| Ho [44] | 12 |
| Ly [45] | 12 |
| Crenshaw [46] | 12 |
| Maristany [53] | 11 |
| Leyerzapf [54] | 12 |
| Osseo-Asare [55] | 12 |
| Kristoffersson [56] | 12 |
| Bullock [57] | 12 |
